# Chitosan coating as an antibacterial surface for biomedical applications

**DOI:** 10.1371/journal.pone.0189537

**Published:** 2017-12-13

**Authors:** Mélanie D’Almeida, Nina Attik, Julien Amalric, Céline Brunon, François Renaud, Hazem Abouelleil, Bérangère Toury, Brigitte Grosgogeat

**Affiliations:** 1 Université Lyon, Université Claude Bernard Lyon 1, CNRS, Laboratoire des Multimatériaux et Interfaces, Villeurbanne, France; 2 Université Lyon, Université Claude Bernard Lyon 1, UFR d’Odontologie, Lyon, France; 3 Science et Surface, Ecully, France; 4 Université Lyon, Université Claude Bernard Lyon 1, CNRS, MATEIS (UMR 5510), Villeurbanne, France; 5 Service de Traitements et de Consultations Dentaires, Hospices Civils de Lyon, Lyon, France; Universite de Technologie de Compiegne, FRANCE

## Abstract

**Background and objectives:**

A current public health issue is preventing post-surgical complications by designing antibacterial implants. To achieve this goal, in this study we evaluated the antibacterial activity of an animal-free chitosan grafted onto a titanium alloy.

**Methods:**

Animal-free chitosan binding on the substrate was performed by covalent link via a two-step process using TriEthoxySilylPropyl Succinic Anhydride (TESPSA) as the coupling agent. All grafting steps were studied and validated by means of X-ray Photoelectron Spectroscopy (XPS), Time-of-Flight secondary ion mass spectrometry (ToF-SIMS) analyses and Dynamic-mode Secondary Ion Mass Spectrometry (DSIMS). The antibacterial activity against *Escherichia coli* and *Staphylococcus aureus* strains of the developed coating was assessed using the number of colony forming units (CFU).

**Results:**

XPS showed a significant increase in the C and N atomic percentages assigned to the presence of chitosan. A thick layer of polymer deposit was detected by ToF-SIMS and the results obtained by DSIMS measurements are in agreement with ToF-SIMS and XPS analyses and confirms that the coating synthesis was a success. The developed coating was active against both gram negative and gram positive tested bacteria.

**Conclusion:**

The success of the chitosan immobilization was proven using the surface characterization techniques applied in this study. The coating was found to be effective against *Escherichia coli* and *Staphylococcus aureus* strains.

## Introduction

In recent years, the development and application of biomaterials has greatly increased in several medical fields such as plastic surgery, ophthalmology, orthopedic and cardiac surgery, urology and odontology [1]. In order to respond to growing clinical needs, it is essential to offer superior performing new biomaterials. Devices based on titanium alloys are mostly acknowledged for their mechanical properties, their excellent corrosion resistance and their high biocompatibility [2,3]. Nevertheless, titanium implant surfaces do not prevent bacterial adhesion and proliferation [4]. The main challenge is to provide antibacterial properties to titanium alloy based implants in order to inhibit bacteria growth and/or disrupt biofilm formation on the implant surface. An efficient method therefore would be the functionalization of implant surfaces with natural bioactive polymers to prevent infections [5].

Various strategies based on titanium surface coating are reported to bring antibacterial properties to the implant surface [6–9]. One of the methods comprises of using chitosan (CS) as a natural bioactive biopolymer commonly employed due to its cytocompatibility, its antibacterial properties and its biodegradability [10]. It can be prepared in different forms such as gels, nanoparticles, fibers, membranes or sponges, allowing a large variety of biological and medical applications such as tissue engineering [11,12], wound healing [13–15], drug delivery [16,17] or skin regeneration [18]. This polysaccharide is a copolymer of *N*-acetyl-*D*-glucosamine and *D*-glucosamine units with β binding at position 1 and 4 obtained by heterogeneous deacetylation of chitin [13,19] and is obtained from microorganisms, some fungi and the exoskeletons of crustaceans [20].

In order to bond chitosan to a titanium support, several methods are considered in the literature; such as the dopamine glutaraldehyde method [21], the electrodeposition of chitosan on substrates [22], or the use of catechol functional groups [23]. Another strategy consists of using an organosilane as a coupling agent; such as 3-AminoPropylTriEthoxySilane (APTES) associated with glutaraldehyde or succinic anhydride [24–27] or TriEthoxySilylPropylSuccinic Anhydride (TESPSA) [28]. Which is often selected as an intermediate between biomolecules and titanium due to its inherent antibacterial and biological properties [29].

Recent *in vitro* studies focused on the surface characterization, especially on the interface between an animal-based chitosan coating and metal support using both bulk and outermost surface characterization techniques [27]. Our group demonstrated the cytocompatiblity of grafted animal chitosan using TESPSA as coupling agent in the presence of fibroblasts [28,30]. However, biological and antibacterial behaviors differ according to chitosan characteristics such as origin, acetylating degree, molar mass and the production conditions [31–33]. Moreover, the biopolymer form, whether film, fiber or sponge, also has an impact on the biological properties [34].

In this study, we present the steps of a method used to graft ultra-pure fungi chitosan onto ultra-pure titanium alloy (T_A6V_). To react with the titanium alloy support, TESPSA is employed as a silane source to ensure the formation of a peptide bond between chitosan and the titanium surface. The presence of the chitosan coating is demonstrated using X-ray Photoelectron Spectroscopy (XPS) and Time-of-Flight Secondary Ion Mass Spectrometry (ToF-SIMS) in static and dynamic modes. Furthermore, the effects of the bioactive coating against two common bacterial strains *Escherichia coli* and *Staphylococcus aureus* were evaluated.

## Materials and methods

### Chemical functionalization of titanium alloy

Titanium alloys (T_A6V_) were supplied by Global D (France), pellets of 1cm machined by Phen’X (batch: 14010091) were used and the raw materials identification was HEPTAL (cast: 82706880–78770). The polymer immobilization on titanium surface using TESPSA was performed as previously reported with modifications [28,29]. After the cleaning and oxidizing steps, leading to samples respectively labeled T_A6V_ and PiT_A6V_, the silanation step of the surface was achieved by immersion of the sample in a solution of TriEthoxySilylPropylSuccinic Anhydride (TESPSA) (ABCR, Germany) dissolved in pentane (v/v, 0.1/100) under argon. After 1 hour, the solvent was removed and the samples were then heated at 150°C for 12 hours. The samples were then cleaned in pentane, tetrahydrofuran and dichloromethane by ultrasound for 20 min each (TPiT_A6Vl_). After drying under inert gas, a biopolymer solution, containing 2 wt % chitosan (DA 21.7, Mw 222 000 g/mol, Kitozym, Belgium) and 3% (v/v) acetic acid in deionized water, was grafted on the samples surface by *dip coating* (v = 1 mm/s). The chitosan coated samples were then dried at 80°C for 24 h and labeled CSTPiT_A6V_.

### Surface chemistry characterizations

#### X-ray Photoelectron Spectroscopy (XPS)

Measurements were carried out using a PHI Quantera SXM instrument (Physical Electronics, Chanhassen, USA) equipped with a 180 hemispherical electron energy analyzer and a monochromatized Al K*α* (1486.6 eV) source operated at 15 kV and 4 mA. The analysis spot had a diameter of 200 μm and the detection angle relative to the substrate surface was 45°. Standard deviations were calculated from measurements performed on two different areas. Data were analyzed using the Multipak software (version v.9.6.0, Physical Electronics). The depth probed by XPS analysis is between 5–10 nm.

#### Time-of-Flight secondary ion mass spectrometry (ToF-SIMS)

Measurements were performed using a ToF-SIMS 5 instrument (ION-TOF GmbH, Germany) following analysis and calibration conditions reported in our previous study [27]. A pulsed primary ion source of Bi_3_^+^ was operated at 25 KeV. The scanning area of secondary ions was 100 μm × 100 μm. The depth probed was about the first monolayer in the static mode. Data were analyzed using the *IONTOF Measurement Explorer s*oftware. Standard deviations were calculated from measurements performed on three different areas. The spectra were acquired in high current bunched mode. When required, charge effects were compensated by means of a pulsed electron flood gun (Ek = 20 eV), the primary ion dose density being then 1.25 × 10^12^ Bi_3_^+^/cm^2^. Spectra calibration was achieved using positions of C^-^, C_3_^-^, C_4_^-^ peaks in negative mode, and CH_3_^+^, C_4_H_7_^+^, C_5_H_9_^+^ peaks in positive mode. Spectra comparison was carried out after a normalization of the intensity, proportionally to the total intensity of each spectrum. Pure liquid TESPSA and self-supported chitosan membrane were used as reference samples in order to select characteristic fragments that could be compared with the covalently linked chitosane coating (Table 1).

**Table 1:** Specific positive and negative fragments of TESPSA and chitosan.

	Negative ion	Measured m/z	Positive ion	Measured m/z
**TESPSA**	SiO_2_^-^	59.967	Si^+^	27.975
	SiHO_2_^-^	60.975	SiHO^+^	44.979
	SiHO_3_^-^	76.968	SiH_3_O_2_^+^	62.991
	CHO_2_^-^	44.999	SiH_3_O_3_^+^	78.984
	C_2_H_5_O^-^	45.034	C_3_H_3_O^+^	55.019
**Chitosan**	CHO_2_^-^	44.998	C_2_H_6_NO^+^	60.043
	C_2_H_4_NO^-^	58.034	C_2_H_5_O_2_^+^	61.027
	C_2_H_3_O_2_^-^	59.015	C_5_H_6_NO^+^	96.049
	C_3_H_3_O_2_^-^	71.016	C_5_H_5_O_2_^+^	97.029
	C_3_H_4_NO_2_^-^	86.025	C_4_H_6_NO_2_^+^	100.041
	C_4_H_6_NO_2_^-^	100.045	C_5_H_6_NO_2_^+^	112.039
			C_6_H_10_NO_3_^+^	144.065

#### Dynamic-mode Secondary Ion Mass Spectrometry (DSIMS)

ToF-SIMS 5 instrument (ION-TOF GmbH, Germany) was used with a pulsed primary ion source of Bi_1_^+^ operated at 25 KeV for analysis, the same operating conditions were selected as used in a previous study [27]. Scanning area of secondary ions was 100 μm × 100 μm in the center of a larger sputtered area, in order to minimize edge effects and redeposition linked to the formation of the crater. A primary ion source of O_2_^+^ was operated in non-interlaced mode at 2 KeV for sputtering with sputtered area of 300 μm × 300 μm. Charge effects were compensated by means of a pulsed electron flood gun (Ek = 20 eV). The depth probed is at the nanometer scale in the dynamic mode, with each point of the profiles obtained from a mass spectrum. Ion intensities were plotted *versus* the sputtering time, which was converted into depth after profilometry measurements.

### Antibacterial assessments

Antibacterial activity of chitosan coated samples was assessed *in vitro* against two bacterial strains: Escherichia coli CIP54127 and Staphylococcus aureus CIP483. These strains were selected in order to represent gram-negative and gram-positive bacteria respectively, according to the ISO 22196: 2007 standard method for the measurement of antibacterial activity on plastics and extended to non-porous surfaces in 2011 [35]. The number of colony forming units (CFU) on PCA was counted immediately after inoculation and after 24 h incubation at 37°C for both control and tested samples. Uncoated titanium alloy was used as control. The antibacterial activity A was calculated using the following formula: ***A = (Ut–U0)–(Ct–C0)*** where ‘***U***’ and ‘***C***’ were the average between the logarithmic number of bacteria enumerated after removal from untreated surfaces and coated titanium, respectively; and ‘***t***’ corresponds to the 0 and 24 h inoculation times tested. A sample was considered as active only if A is higher than 1 and bactericidal when the number of viable bacteria is null. All experiments were performed in duplicate with three samples at each (n = 6).

## Results and discussion

### Control of functionalization of titanium alloy surface

#### X-ray Photoelectron Spectroscopy (XPS) analysis

**Fig 1:**
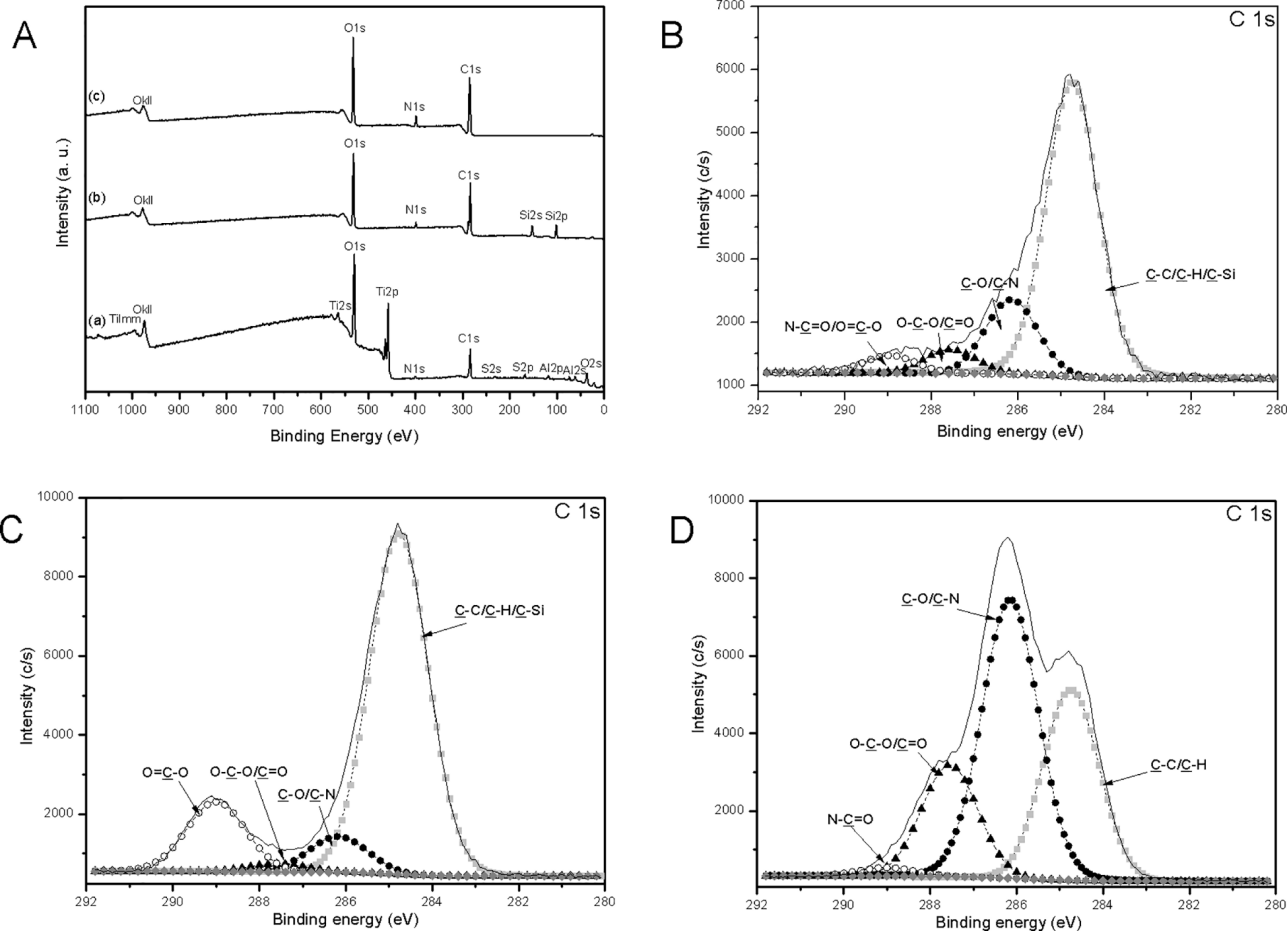
XPS general survey (A) of PiTA6V (a), TPiTA6V (b) and CSTPiTA6V (c) and XPS high resolution and deconvolution of carbon spectra of PiTA6V (B), TPiTA6V (C) and CSTPiTA6V (D).

XPS is considered to be a very highly sensitive method for the characterization of the surface chemistry of materials used in different applications, especially in biotechnology [36] and to verify the chemical composition of different coatings [37,38]. Hence, this technique was used to analyze all samples after grafting. Atomic elemental compositions measured at the surface of all samples are reported in Table 2 and XPS survey spectra of PiT_A6V_ (a), TPiT_A6V_ (b) and CSTPiT_A6V_ (c) are presented in Fig 1A. In addition, deconvolution of the carbon signal was observed in order to allow the differentiation of carbon chemical environment. Changes of chemical bonding nature in the carbon environment allowed the success of the grafting procedure. Fig 1B, 1C and 1D present deconvolutions of the carbon signals detected in samples PiT_A6V_ (B), TPiT_A6V_ (C) and CSTPiT_A6V_ (D).

**Table 2:** Atomic elemental composition percent for the individual reaction steps determined by XPS. The different surfaces obtained were labeled as follow: unmodified surface (T_A6V_), piranha treated titanium alloy surface (PiT_A6V_), TESPSA modified PiT_A6V_ (TPiT_A6V_) and chitosan modified TPiTA6V surface (CSTPiT_A6V_).

	T_A6V_	PiT_A6V_	TPiT_A6V_	CSTPiT_A6V_
**C1s (%)**	34.8 ± 0.9	30.7 ± 0.4	54.1 ± 0.3	61.6 ± 1.4
**N1s (%)**	1.2 ± 0.1	1.6 ± 0.7	2.8 ± 0.3	6.6 ± 0.2
**O1s (%)**	46.9 ± 0.6	49.6 ± 0.3	32.2 ± 0.3	31.8 ± 1.2
**Ti2p (%)**	13.3 ± 0.3	12.7 ± 1.0	0.0 ± 0.0	0.0 ± 0.0
**Si2p (%)**	0.0 ± 0.0	0.0 ± 0.0	10.9 ± 0.7	0.0 ± 0.0
**Al2p (%)**	2.6 ± 0.1	4.2 ± 0.3	0.0 ± 0.0	0.0 ± 0.0
**Others (%)**	1.2 ± 0.2	1.2 ± 0.7	0.0 ± 0.0	0.0 ± 0.0

Comparison between elemental compositions of samples before and after piranha treatment demonstrated the positive effect of the oxidizing step since the oxygen content increased, meaning more Ti-OH sites were available at the sample surface. The relative content of atomic ratios of O/Ti of T_A6V_ (3.53 ± 0.03) and PiT_A6V_ (3.92 ± 0.32) samples were in agreement with these results. In the same way, a decrease in the carbon content may imply less surface contamination. Nevertheless, as previously noticed, it appears difficult to eliminate all contaminations, partially due to the high surface reactivity and atmospheric elements [25].

The silane grafting is highlighted by a significant increase of the C and Si atomic percentages. Meanwhile, absence of Ti peak is in agreement with a total masking of the surface after the silanation step (Fig 1A). These observations are in accordance with the total covering of the sample surface with a silane layer over 10 nm (XPS detection limit). On TPiT_A6V_, the C peak was deconvoluted into three main components at 289 eV (15.4 ± 0.6%), at 286.2 eV (6.5 ± 0.8%) and at 284.8 eV (77.3 ± 2.3%) which were attributed to O = C-O, C-O/C-N and C-C/C-H/C-Si respectively and the last component at 287.6 eV (0.8 ± 0.9%) was attributed to O-C-O/C = O. Therefore, due to the non-detection of silicium on PiT_A6V_ sample in the general survey, the signal at 284.8 eV was assigned to C-C or C-H chemical groups. On TPiT_A6V_ sample, an increase in this signal (Fig 1C) could be characteristic of the new C-Si environment brought by the silane. Here the silane signature and the masking of substrate were present still after all the washing steps. This result proved the strong link between titanium alloy and silane. In the same way, the signal at 289.0 eV was attributed to O = C-O environment characterizes the anhydride function of the silane. The effective covalent grafting of silane onto titanium-based support was previously observed [29].

Finally, following the biopolymer deposition step (CSTPiT_A6V_), a significant increasing of C and N atomic percentages assigned to the chitosan could be observed. In parallel, no detection of Ti and Si peaks are in agreement with the total masking of the silane. The deconvoluted peaks of C 1s gave four components: components at 289 eV (2.0 ± 0.1%), at 287.6 eV (15.9 ± 1.5%), at 286.2 eV (51.5 ± 2.8%) and at 284.8 eV (30.7± 4.0%) which were assigned to N-C = O, O-C-O/C = O, C-O/C-N and C-C/C-H respectively [39,40]. These results confirm the success of chitosan grafting as previously noticed [27,28].

#### Time-of-Flight secondary ion mass spectrometry (ToF-SIMS)

**Fig 2:**
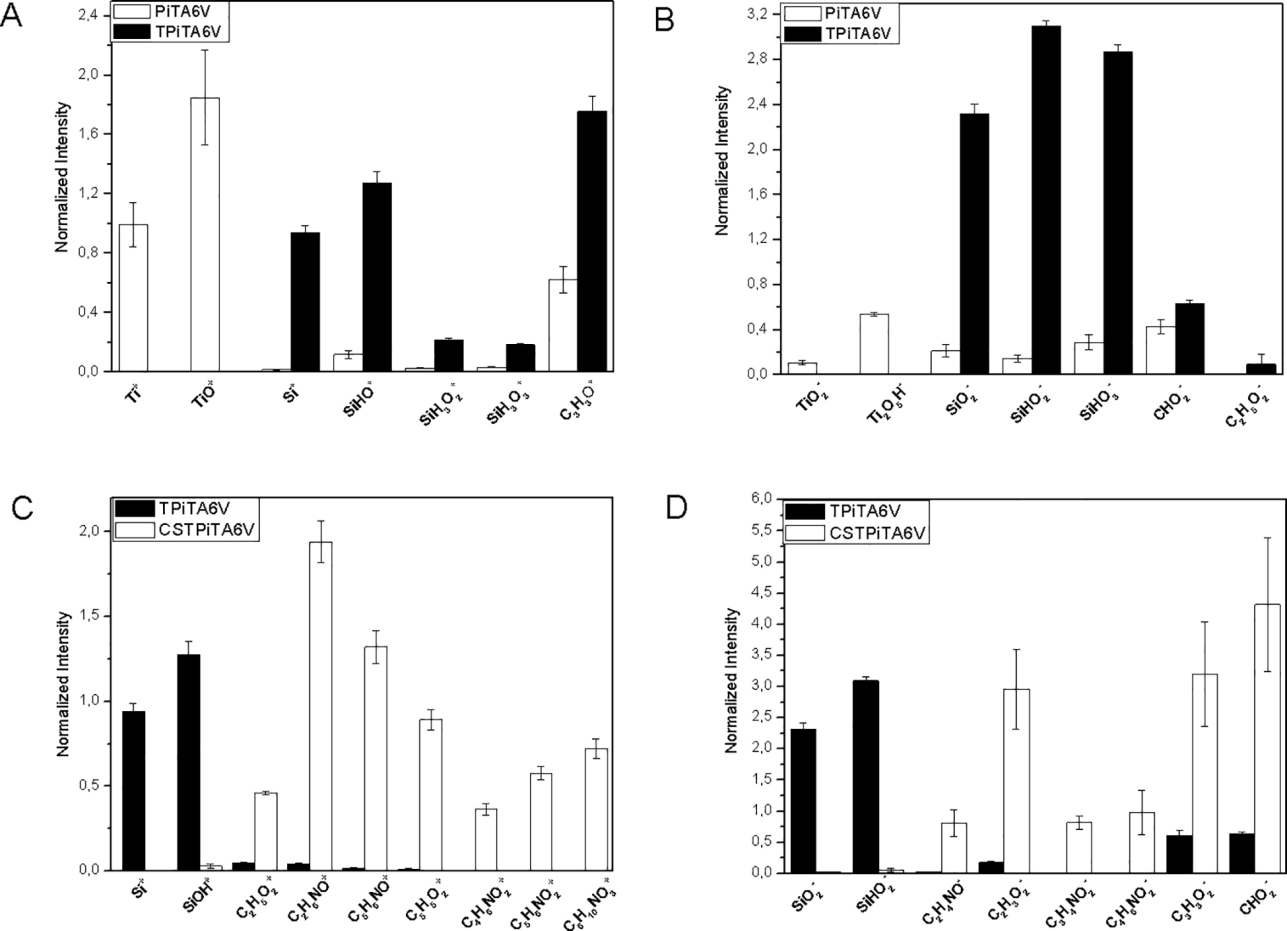
Average normalized intensity of positive and negative fragments of PiT_A6V_ and TPiT_A6V_ (A) and (B) respectively, TPiT_A6V_ and CSTPiT_A6V_ (C) and (D).

Analyses of positive and negative fragments allowed the monitoring of each step of the grafting process to the support (Fig 2). Following the silanation step, characteristic positive and negative fragments of TESPSA were investigated (Fig 2A and 2B) and compared to the uncoated sample PiT_A6V_. Results showed the complete masking of the substrate after silane deposition, proven by the decreasing amounts of Ti^+^, TiO^+^, TiO_2_^-^ and Ti_2_O_5_H^-^. In addition, absence of molecular fragments corresponding to TESPSA alone such as SiC_13_H_25_O_6_^+^ [M+H]^+^ and SiC_13_H_25_O_7_^-^ [M+OH] were in agreement with the effective reaction between the support and the silane. A cross-polymerization reaction could occur and strengthen the film *via* the Si-O-Si bond formation.

After chitosan deposition, characteristic positive and negative fragments of biopolymer defined previously were fully detected (Fig 2C and 2D) [27,41]. In parallel, the decrease of silane fragment intensities suggested a thick layer of deposited polymer. XPS and ToF-SIMS analyses proved the efficiency of chitosan deposition. However, it seems necessary to study the interface between each layer after the coating synthesis.

#### DSIMS analysis

**Fig 3:**
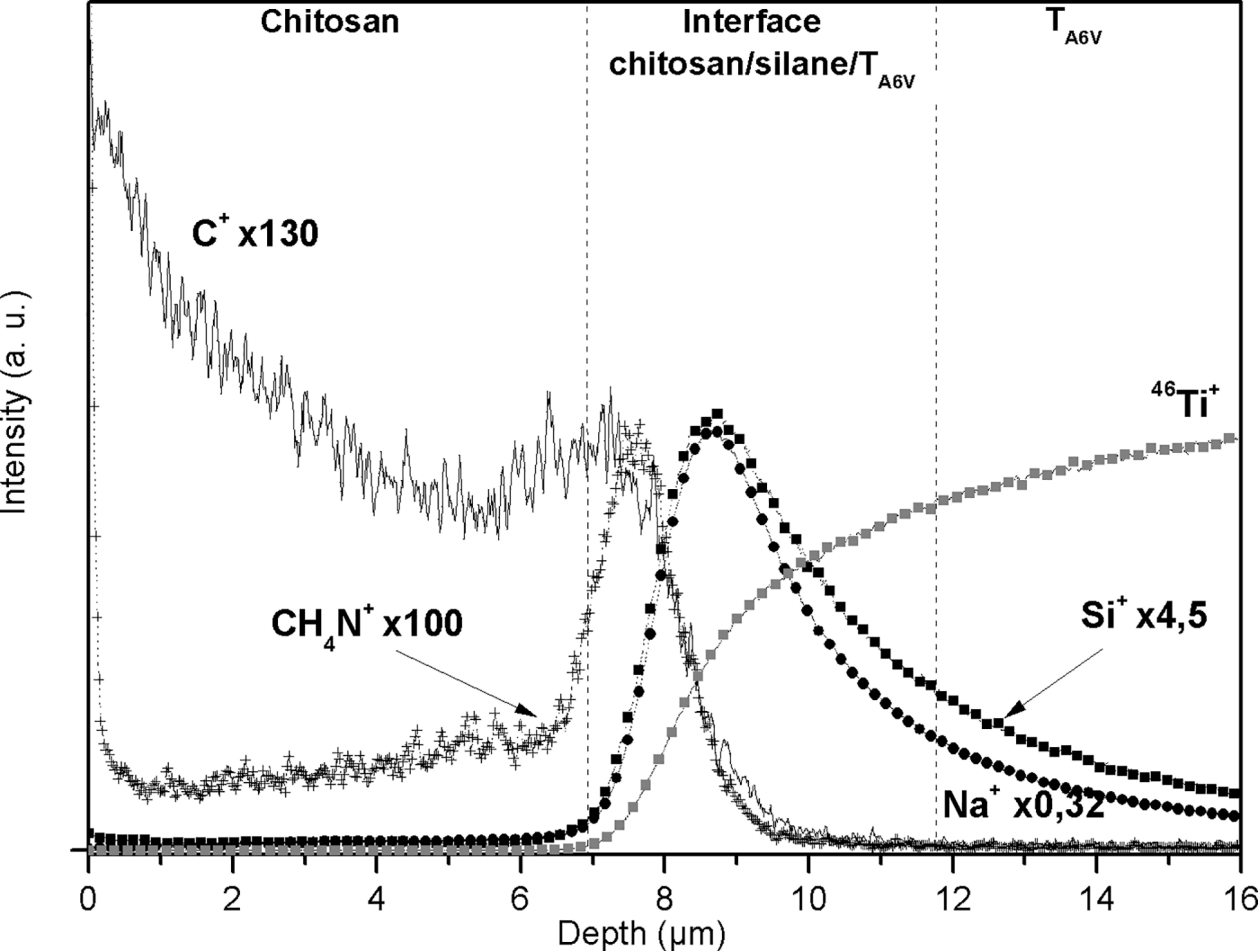
ToF-SIMS depth profiles of positive fragments recorded from chitosan coated sample (CSTPiTA6V).

The results obtained by DSIMS measurements are in agreement with ToF-SIMS and XPS analyses and confirms the coating synthesis success. Each step of the process was characterized using DSIMS analysis [39]. ToF-SIMS positive ion depth profiles were recorded on chitosan-coated samples after completion of the process (Fig 3). As previously reported, the distribution of ^46^Ti^+^, C^+^, Si^+^, CH_4_N^+^ and Na^+^ ion intensities were profiled using a linear scale [27]. Three successive regions were observed on the CSAPiT_A6V_ sample. The presence of C^+^ and CH_4_N^+^ was assigned to the presence of biopolymer on the support. The C^+^ fragment, a characteristic signal of the organic layer, decreased until a second area attributed to the polymer/silane/substrate interface became prominent. This second area was characterized by the presence of Si^+^ ions which corresponded to the TESPSA layer and by the enrichment and disappearance of CH_4_N^+^. Therefore, at this point, the bond formation between chitosan and silane also was suggested. Alkaline contaminations like Na^+^ ions were found at the organic and the interface layer. In parallel to the decrease of Si^+^ fragments, ^46^Ti^+^ fragments increased in the last area assigned to the support.

### Antibacterial activity

The antibacterial properties of chitosan-coated titanium samples were evaluated by measurements of number of CFU (colony-forming unit). The results obtained for polymer-coated titanium samples versus uncoated samples against *E*. *coli* and *S*. *aureus* bacteria strains are detailed in Table 3 (Table 3). As expected, uncoated titanium samples had no antibacterial activity after 24 hours of exposure. On the other hand, no viable bacteria were found in the presence of TESPSA-chitosan titanium samples after 24 hours of contact. In the current experimental conditions, chitosan-coated samples showed effective bactericidal potential against the two strains tested.

**Table 3:** Antibacterial activity of uncoated and chitosan coated samples after 24 h of direct contact.

Samples	Viable *E*. *coli* (CFU/cm^2^)	Viable *S*. *aureus* (CFU/cm^2^)	Log CFU/cm^2^		Antibacterial activity (Log CFU/cm^2^)	
			*E*. *coli*	*S*. *aureus*	*E*. *coli*	*S*. *aureus*
**After inoculation (t = 0)**						
**T**_**A6V**_	(1.73 ± 0.03)× 10^4^	(1.16 ± 0.17) × 10^4^	4.24 ± 0.08	4.06 ± 0.07	No activity	No activity
**CSTPiT**_**A6V**_	(3.65 ± 2.4) × 10^2^	(5.73 ± 3.44) × 10^1^	2.56 ± 0.30	1.76 ± 0.24	No activity	No activity
**After 24 h of contact**						
**T**_**A6V**_	(1.67 ± 0.05)× 10^6^	(1.31 ± 0.77) × 10^5^	6.22 ±0.01	5.12 ± 0.24	No activity	No activity
**CSTPiT**_**A6V**_	0	0	-	-	Bactericide	Bactericide

Biofilm formation occurs after the implantation of medical devices on either hard or soft tissues, and is the major cause of implant failure and bacterial infections. It is recently reported that infections and inflammation are the most common forms of postoperative complications in the case of implantable titanium-based biomaterials. Because of the biocompatibility of titanium surface, titanium implants are suitable substrates for microbial colonization and biofilm formation, which is still a serious clinical problem [42,43]. The local and systemic application of antibiotics is the most common method used to treat and prevent bacterial infections. However, increased resistance to antibiotics in different bacterial strains is the main concern related to this type of therapy and was recently considered as a “global threat” to human health [44].

The development of biomaterials with antibacterial properties is the ultimate goal to decrease disease occurrence and improve health. In this context, chitosan immobilization on a titanium surface using TESPSA process was performed to obtain antibacterial activity of the implant tested. The developed chitosan-coated implant was able to inhibit *E*. *coli* and *S*. *aureus* bacteria growth; these two bacteria strains are commonly tested when the antibacterial ability of an implant coating has to be assessed [45]. In the present study, the chemical process TESPSA was used and did not affect the natural antibacterial properties of the animal-free chitosan used. The developed chitosan-coated implant proved to be an excellent bacterial agent against representative gram-positive and gram-negative bacteria. Furthermore, the use of high-purity non-animal chitosan should be highlighted in the current study as it is reported to be non-allergenic and better tolerated by the human organism which could make it easier to bring the developed medical devices to the market.

## Conclusion

A covalent grafting of fungi chitosan to medical grade TA6V substrate was performed using a two-step process. The coupling agent TESPSA that presents an anhydride function was selected since it is commonly used in biomedical applications. The success of the chitosan immobilization was proved using the surface characterization techniques applied. An ideal coating for biomedical applications should have antibacterial activity against bacterial strains found in the implant vicinity, limiting bacterial adhesion and proliferation and therefore preventing implant-related infections. Furthermore, and due to its chemical resistance under acidic conditions, this chitosan coating design demonstrates potential as an antibacterial coating for different medical devices. This study represents one of the steps before further investigation for clinical use.
